# Chronic pain associated with the Chikungunya Fever: long lasting burden of an acute illness

**DOI:** 10.1186/1471-2334-10-31

**Published:** 2010-02-19

**Authors:** Daniel Ciampi de Andrade, Sylvain Jean, Pierre Clavelou, Radhouane Dallel, Didier Bouhassira

**Affiliations:** 1 INSERM, U-987 Boulogne-Billancourt, F-92100 France; CHU Ambroise Paré, Centre d'Evaluation et de Traitement de la Douleur, AP-HP, Boulogne-Billancourt, F-92100 France; 2 Chemin Maunier, 97440 Saint-André, La Réunion France; 3 INSERM, U929, Clermont-Ferrand, F-63000 France; Université Clermont1, Clermont-Ferrand, F-63000 France; CHU Clermont-Ferrand, Clermont-Ferrand, F-63000 France

## Abstract

**Background:**

Chikungunya virus (CHIKV) is responsible for major epidemics worldwide. Autochthonous cases were recently reported in several European countries. Acute infection is thought to be monophasic. However reports on chronic pain related to CHIKV infection have been made. In particular, the fact that many of these patients do not respond well to usual analgesics suggests that the nature of chronic pain may be not only nociceptive but also neuropathic. Neuropathic pain syndromes require specific treatment and the identification of neuropathic characteristics (NC) in a pain syndrome is a major step towards pain control.

**Methods:**

We carried out a cross-sectional study at the end of the major two-wave outbreak lasting 17 months in Réunion Island. We assessed pain in 106 patients seeking general practitioners with confirmed infection with the CHIK virus, and evaluated its impact on quality of life (QoL).

**Results:**

The mean intensity of pain on the visual-analogical scale (VAS) was 5.8 ± 2.1, and its mean duration was 89 ± 2 days. Fifty-six patients fulfilled the definition of chronic pain. Pain had NC in 18.9% according to the DN4 questionnaire. Conversely, about two thirds (65%) of patients with NC had chronic pain. The average pain intensity was similar between patients with or without NC (6.0 ± 1.7 vs 6.1 ± 2.0). However, the total score of the Short Form-McGill Pain Questionnaire (SF-MPQ)(15.5 ± 5.2 vs 11.6 ± 5.2; p < 0.01) and both the affective (18.8 ± 6.2 vs 13.4 ± 6.7; p < 0.01) and sensory subscores (34.3 ± 10.7 vs 25.0 ± 9.9; p < 0.01) were significantly higher in patients with NC. The mean pain interference in life activities calculated from the Brief Pain Inventory (BPI) was significantly higher in patients with chronic pain than in patients without it (6.8 ± 1.9 vs 5.9 ± 1.9, p < 0.05). This score was also significantly higher in patients with NC than in those without such a feature (7.2 ± 1.5 vs 6.1 ± 1.9, p < 0.05).

**Conclusions:**

There exists a specific chronic pain condition associated to CHIKV. Pain with NC seems to be associated with more aggressive clinical picture, more intense impact in QoL and more challenging pharmacological treatment.

## Background

Chikungunya fever is a viral disease caused by the arthropod-borne Chikungunya virus, from the *Togaviridae *family. It is transmitted to humans by the *Aedes *ssp mosquitoes. The virus was first isolated in 1953, in Uganda, during an epidemic in the province of Newala in Tanganyika (now Tanzania)[1]. Its name derives from the Makonde language, meaning, "he, who walks bent up". The infection gives rise to an unusual clinical finding during its clinical curse: pain, which is virtually universal and is the major symptom of the disease. Since the recognition of the first case of CHIKV infection, both sporadic and major epidemics have been reported in Africa, India, South-East Asia and Western Pacific [1,2]. Epidemics of unprecedented magnitude occurred in 2005-2006 in the islands of the South-West Indian Ocean. In particular, there was a major outbreak of CHIKV in Réunion Island, a French overseas *département *(French administrative unit), with 266,000 people infected, constituting 34% of the island's total population [3].

Interest in this viral infection has grown in recent years. Competent vectors (i.e. *Aedes *mosquitoes) are widely distributed throughout the world; thus many countries not initially hit by previous epidemics present a potential risk of outbreaks [4]. Autochthonous cases were recently reported in northern Italy [5] and imported cases in travelers returning from affected areas have been reported in several European countries and in the United States of America [6-8]

The clinical course of the acute phase of infection has been well characterized during previous epidemics in African countries, the Indian subcontinent and Southeast Asia [1]. It involves an incubation period lasting between two to six days, followed by the abrupt onset of fever associated with intense diffuse muscle and joint pain. Headache, photophobia, nausea, vomiting, diarrhea and a maculopapular or morbilliform skin rash may accompany these symptoms. Treatment is mainly symptomatic, with remission observed in most patients some days after the infection.

Some studies have reported cases in which arthralgia persists after resolution of the acute infection, thus leading to chronic pain [9,10]. Despite these initial studies, a broader characterization of chronic pain related to Chikungunya infection is still needed. In particular, the fact that many of these patients do not respond well to usual analgesics suggests that the nature of chronic pain may be not only nociceptive but also neuropathic. The neurotropism of CHIK virus, reflected in the neurological complications [11], is compatible with this notion. Given that neuropathic pain syndromes require specific treatment, including antiepileptics and antidepressants [12], the potential neuropathic component of this disease should be a major factor affecting the symptomatic management of such cases.

We carried out a study at the end of the major two-wave outbreak lasting 17 months in Réunion Island. The aims of this study were to assess and characterize pain, particularly chronic pain in patients attending general practices who have confirmed serologic infection by the CHIKV and to evaluate the impact of this pain on quality of life (QoL).

## Methods

This study was approved by our institutions ethics review board in compliance to the Helsinki declaration. It was carried out from June to July 2006 in 13 general practices located throughout Réunion Island. All subjects received written information on the study and gave written informed consent prior to participation. All patients spontaneously seeking medical attention were directly asked whether they were on pain. Patients were included if they presented with any type of pain and previous serologic confirmation of CHIKV infection based on CHIKV IgG and IgM detected by direct ELISA and ELISA following immunocapture, respectively, as originally established at the Centre National de Référence pour les Arbovirus (Pasteur Institute, Lyon, France) [13]. The enrollment period for the study lasted five consecutive working days, and each patient was seen only once. Patients presenting with signs of severe disease such as meningismus, intense headaches or hemodynamic instability were headed to the nearest hospital and were not included in the study. We excluded patients presenting with pain clearly related to any other etiologies (i.e. rheumatologic, muscular, neurological - i.e: migraines), or those presenting with diabetes, psychiatric illness or a history of drug abuse, including alcohol, since all these conditions may be associated to specific painful syndromes and thus might constitute confounders to our analysis. The assessment included a structured interview and specific questionnaires to characterize the painful syndrome and its impact on QoL [14]. The study was approved by our institution's review board and all patients signed a written informed consent.

The intensity, location and effect of pain on quality of life were assessed with the Brief Pain Inventory (BPI) [14]. In this largely used questionnaire, three numerical rating scales each ranging from 0 (no pain) to 10 (maximal pain) were used to assess minimal, maximal and average pain intensity over the previous week. Then patients were asked to report all sites of pain on a diagram of the body and to specify the location of the most intense pain. The BPI also includes a series of numerical rating scales (equally ranging from 0 to 10) to assess the extent that pain interferes with general activity, mood, walking, sleep, work, relationship with others and life enjoyment (from 0: does not interfere, to 10: complete interference). The short-form McGill Pain Questionnaire (SF-MPQ) [15] was used to measure the sensory and affective dimensions of pain. This tool has been widely used in pain studies. It comprises 15 descriptors (11 sensory; 4 affective) which are rated on an intensity scale as 0 = none, 1 = mild, 2 = moderate or 3 = severe. Three pain scores are derived from the sum of the intensity rank values of the words chosen for sensory, affective and total descriptors. The DN4-interview questionnaire, includes seven pain descriptors that must be answered ¨yes¨ or ¨no¨ based on their presence or absence, respectively, in a given painful body location [16]. Since this questionnaire is aimed at differentiating neuropathic pain from other pain syndromes (ie.: nociceptive pain) all its descriptors must be directed to the same painful phenomenon at a time (ie.: the same body region). Quite frequently, patients are asked to answer the questionnaire according to the site of the ¨most troublesome pain¨. So patients with diffuse pain, but presenting a body region where pain is more intense can easily respond to the questionnaire. This method has been shown to present a high sensibility and specificity in diverse pain trials [12]. However, it is recommended that it should not be administered to patients who have a similar intensity of pain in multiple locations. The duration of pain was also assessed and chronic pain was defined as daily pain for more than three months [17].

In addition, patients were asked to provide details of current pain treatment (eg.: analgesic drugs prescribed so far) and their efficacy: low = < 30% pain reduction, moderate = 30-70% pain reduction and good = > 70% pain reduction. Pain relief after analgesics was considered a >30% pain reduction from baseline [12].

### Statistical Analysis

Quantitative variables were expressed as means and standard deviations (SD). Qualitative variables expressed as proportions and percentages. We used analyses of variance (ANOVA), with Fisher's PLSD test, to compare pain intensity and duration and questionnaire scores between patients with or without chronic pain and between patients with or without neuropathic characteristics. Multiple regression was used to analyze the association between the effect of pain on QoL (i.e. mean interference score) and clinical characteristics (i.e. age, pain intensity and duration, DN4 questionnaire score, SF-McGill sensory and affective scores). The Chi^2 ^test was used to compare proportions. P < 0.05 was considered significant in all instances.

## Results

### Pain characteristics

One hundred and six consecutive painful patients (79 women) were included in this study (47.3 ± 11.9 years). All reported pain at multiple sites. All patients reported pain in at least one joint (Table 1). Besides joint pain, some patients also reported nonarticular pain, especially on the lower limbs.

**Table 1:** Description of the patients and percentages of patients reporting pain at different locations

Clinical and demographic data	
Mean age ± SD (range)	47.33 ± 11.9 (19-73)
Sex (women/men) (%)	74.5/25.5
Mean duration of pain (days ± SD) (range)	89.1 ± 55.5 (1-318)
Mean pain intensity ± SD (range)	5.8 ± 2.1 (1-10)

***Pain locations***	***%***
Head/neck	26
Thorax/abdomen	14
Back	49
Upper Limbs	95
Lower Limbs	98
*Location of joint pain*	
Shoulder	54
Elbow	48
Hand/wrist	77
Hip	9
Knee	72
Ankle/foot	81

The mean intensity of pain measured by the BPI was 5.8 ± 2.1, with minimum and maximum pain intensity of 3.6 ± 2.1 and 8.3 ± 1.9, respectively.

The mean duration of pain was 89 ± 2 days. Seventeen patients (16%) had suffered pain for less than one month, 33 patients (31%) between one and three months and 56 (53%) patients suffered chronic pain (mean: 128 ± 41 days, range: 95-318 days).

Pain had neuropathic characteristics (NC) in 20 patients (18.9%) of patients. Pain with NC was located mostly in the upper (37%) or lower (48%) limbs and more rarely in the back (7%) or head/neck (7%).

About half (53%) of patients with NC had chronic pain. The average pain intensity was similar between patients with or without NC (6.0 ± 1.7 vs 6.1 ± 2.0). However, the total score of the SF-MPQ (15.5 ± 5.2 vs 11.6 ± 5.2; p < 0.01) and both the affective (18.8 ± 6.2 vs 13.4 ± 6.7; p < 0.01) and sensory subscores (34.3 ± 10.7 vs 25.0 ± 9.9; p < 0.01) were significantly higher in patients with NC.

### Impact on quality of life

The mean pain interference score calculated from the BPI was significantly higher in patients with chronic pain than in patients without (6.8 ± 1.9 vs 5.9 ± 1.9, p < 0.05). This score was also significantly higher in patients with NC than in those without NC (7.2 ± 1.5 vs 6.1 ± 1.9, p < 0.05).

**Figure 1:**
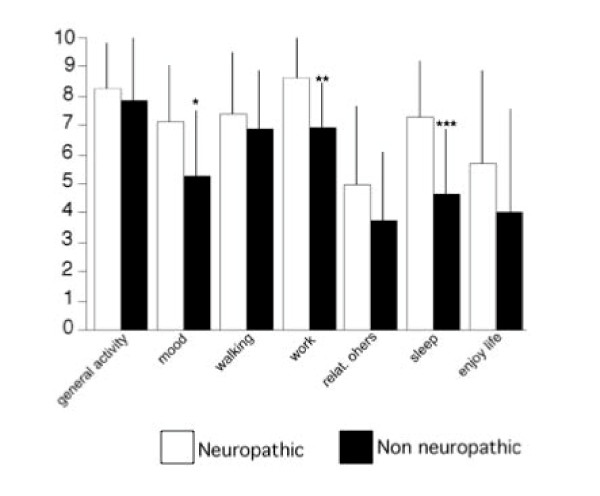
**Comparison of BPI items related to the impact ("interference") of pain on quality of life, between patients with pain with or without neuropathic characteristics**. Abbreviations: relat. others: relationship with others. * p < 0.05; **p < 0.01; ***p < 0.001

Multiple regression analysis showed that the interference of pain with quality of life was significantly associated with average pain intensity, the DN4 score and SF-MPQ affective score, but not with age or duration of pain (Table 2). The items altered the most were working, mood and sleep (Figure 1).

**Table 2:** Results of the multiple regression analysis showing the association between alteration of mean pain interference score and mean pain intensity, SF-McGill affective and sensory scores and DN4 questionnaire score

	Coefficient	Standardized Error	Standardized Coefficient	t	p
age	0.017	0.011	0.107	1.525	0.130
Pain duration	0.001	0.003	0.019	0.271	0.787
Mean pain intensity	0.233	0.069	0.246	3.352	0.0011
SF-McGill sensory	-0.51	0.027	-0.154	-1.855	0.066
SF-McGill affective	0.139	0.024	0.495	5.723	<0.0001
DN4 score	0.386	0.088	0.323	4.362	<0.001

### Symptomatic treatment

Eighty-eight (83%) patients had received analgesic treatment for their pain. The most frequently used drugs included: corticoids (40%), non-steroid anti-inflammatory drugs (NSAID) (22%) and acetaminophen (8%) (Table 3). Twenty-five (24%) patients reported poor pain relief, 53 patients (50%) reported moderate pain relief and 28 (26%) good pain relief with their symptomatic treatment. The percentage of pain relief was significantly lower (p < 0.01) in patients with NC (39 ± 24%) than in patients without NC (56 ± 27%).

**Table 3:** Pain treatment used for CHIKF-related pain and number (%) of patients under treatment

Main analgesic treatment	N = 106 (%)
Corticosteroids	42 (40%)
Non-steroidal anti-inflammatory drugs	23 (22%)
None	18 (17%)
Acetaminophen	9 (8%)
Dextropropoxiphen	6 (6%)
Physical therapy	2 (2%)
Acetaminophen + opioid	1 (1%)
Tricyclic antidepressants	1 (1%)
Acupuncture	1 (1%)
Antiepileptic drugs	1 (1%)
Tramadol	1 (1%)

## Discussion

Our data from a sample of painful patients attending general practices presenting with pain and serologically confirmed infection by the CHIKV after the last major CHIKV outbreak in Réunion Island showed that about half (51%) of these patients suffered from chronic pain. Consistent with previous reports, all of our patients presented with arthralgia. Joint involvement has been increasingly recognized in CHIKV infections and its relationship with certain predisposing genetic profiles, such as HLA B27, has been proposed [18-20]. However, we identified a subgroup of patients whose most troublesome pain was not located in the joints and had neuropathic characteristics (NC) (ie.:, burning pain, cold pain, electric-shoks like pain, tingling, pins and needles, numbness, itching,). The presence of NC, related with specific pain mechanisms, was associated with a significantly poorer quality of life and lower efficacy of treatment.

The association between chronic pain and CHIKV has been assessed in only a few studies [9,10]. The largest retrospective study from Réunion Island suggested that up to 63% of hospitalized patients are affected by chronic joint pain caused or aggravated by CHIKV infection [10]. Previous studies focused on chronic arthralgia and it was suggested that a rheumatoid syndrome could be responsible for chronic pain in these patients. Consistent with this, Brighton and co-workers [9] found high antibody titers against CHIKV in the synovial fluid of patients with persistent joint pain and rigidity (5.6% of patients studied). In our study, although chronic arthralgia was ubiquitously present, patients also reported pain in other locations. The mechanisms of nonarticular chronic pain associated with CHIKV infection are still poorly understood and remains elusive. Autopsy studies in other neuroinfectious syndromes, such as zoster radiculopathy, have shown that even years after the viral reactivation axonal atrophy and loss of myelin in peripheral nerves may still be detected. Also, some major pathological changes, such as dorsal horn atrophy were present only in those patients presenting chronic pain after the viral reactivation (post-herpetic neuralgia) [21,22] This argues for the fact that an active pathological process may take place after the acute infection, being associated with long term pain symptomatology. About one out of five of our patients reported pain with NC, suggesting that other mechanisms were also involved. Neuropathic pain syndromes are caused by a lesion or dysfunction of the nervous system and their mechanisms are not completely understood. However, it is well established that neuropathic pain syndromes do not depend directly on inflammatory processes, but involve specific peripheral and central changes in nociceptive processes [12,16]. The fact that our patients did not present with obvious peripheral or central neurological findings may indicate that NC reflected a dysfunction of the nervous system, rather than a neurological lesion induced by the CHIKV. Further clinical and experimental studies will be needed to identify the putative (peripheral and/or central) neurological lesion or dysfunction in these patients.

Nevertheless, this subgroup of patients deserves particular attention. Indeed, the presence of NC was associated with a less favorable outcome, in terms of a greater impact on quality of life and lower efficacy of treatment. In particular, the poorer outcome of treatment may be explained by the fact that neuropathic pain syndromes, which do not respond to conventional analgesics, respond better to antiepileptics and tricyclic antidepressants [12], which were used in only a minority of our patients.

Whether our findings could be extrapolated to CHIKV infections in other geographic areas remains uncertain, since a series of findings suggest that CHIKV infection during the Réunion outbreak was particularly aggressive. Thus, more cases with severe neurological complications, such as meningoencephalitis, requiring intensive-care units, and the first cases of vertical maternal fetal transmission were reported during this outbreak [1-4,23,24]. New viral mutations, not detected during previous epidemics, were detected during this outbreak and may thus be related to the more aggressive clinical progression of the infection [1]. Nevertheless, CHIKV strains isolated during Réunion Island epidemics show 99.61% homology to strains isolated in India [25]. It is plausible that such an aggressive infection profile will affect other regions during future outbreaks [26]. Also, we found a greater proportion of women with pain symptoms, which has also been found in large epidemiological studies [27]. Although a female predisposition to present pain after CHIKV infection cannot be ruled out, it could also be related to differences in access to primary care between men and women. Other studies are needed to address this question.

Another limitation is that our study was based on the evaluation of patients in a primary care setting. Although this allowed a broad assessment of the study population, more severe cases, treated in secondary and tertiary centers, may have been missed. We also only evaluated patients with painful syndromes seeking medical attention and therefore could not estimate the actual prevalence of chronic pain in CHIKV infections. Future prospective studies including long-term follow-up are warranted to estimate the prevalence of chronic pain related to CHIKV infections.

## Conclusions

This study suggests that chronic pain is a frequent long-term complication of CHIKV and that the presence of neuropathic features is associated with a more severe condition. Thus, although its mechanisms remain to be determined, improved characterization of pain, including the identification of neuropathic features with an easy-to-use clinical tool, could help to significantly improve treatment outcome.

## Competing interests

The authors declare that they have no competing interests.

## Authors' contributions

DCA and DB have made substantial contributions to conception of the study, data analyses, and writing the draft of the manuscript. SJ, PC, and RD contributed to data acquisition, analysis and interpretation. DB was involved in revising the manuscript critically for important intellectual content. All authors contributed to the writing of the manuscript, and all approved its final version.

## Pre-publication history

The pre-publication history for this paper can be accessed here:

http://www.biomedcentral.com/1471-2334/10/31/prepub
